# Role of eIF4A1 in triple‐negative breast cancer stem‐like cell‐mediated drug resistance

**DOI:** 10.1002/cnr2.1299

**Published:** 2020-10-14

**Authors:** Dayanidhi Raman, Amit K. Tiwari

**Affiliations:** ^1^ Department of Cancer Biology University of Toledo Health Science Campus Toledo Ohio; ^2^ Department of Pharmacology & Experimental Therapeutics University of Toledo Health Science Campus Toledo Ohio

**Keywords:** ABC transporters, breast cancer stemness, chemoresistance, eIF4A1, triple‐negative breast cancer

## Abstract

In cap‐dependent translation, the eukaryotic translation initiation factor 4A (eIF4A1) is an mRNA helicase is involved in unwinding of the secondary structure, such as the stem‐loops, at the 5′‐leader regions of the key oncogenic mRNAs. This facilitates ribosomal scanning and translation of the oncogenic mRNAs. eIF4A1 has a regulatory role in translating many oncoproteins that have vital roles in several steps of metastases. Sridharan *et. al*. have discovered and provide a novel insight into how eIF4A1 can play a regulatory role in drug resistance by influencing the levels of pluripotent Yamanaka transcription factors and ATP‐binding cassette (ABC) transporters in triple‐negative breast cancer (TNBC) stem‐like cells. These findings may help us understand the molecular underpinnings of chemoresistance, especially in established metastases in TNBC. Importantly, eIF4A1 may form a novel clinical target in metastatic TNBC and the drug eFT226 from Effector Therapeutics targeting eIF4A1 is already in phase1‐2 clinical trial.

Triple‐negative breast cancer (TNBC), defined by the lack of estrogen (ER) and progesterone receptors (PR), and the absence of human epidermal growth factor receptor 2 (HER2) overexpression, often leads to high‐grade invasive ductal carcinoma (IDC) in the patients accounting for one‐fourth of all breast cancer deaths.^1^, ^2^ Although the prevalence of TNBC is only around 15%, the metastatic nature of TNBC results in poor clinical outcome in majority of TNBC patients. Furthermore, the median overall survival (OS) in metastatic TNBC (mTNBC) is around 18 months, whereas in the luminal breast cancer cases, expressing ER/PR or HER2, it exceed 5 years. It affects more of the premenopausal and young African American women. Following standard platinum/taxane/anthracycline neoadjuvant chemotherapy (NACT) in TNBC patients, there is an initial response but followed by an increased rate of relapse frequently accompanied by distant metastases (TNBC paradox). The pathological complete response (pCR) to FDA‐approved targeted therapy against poly ADP ribose polymerase (PARP) in TNBC patients is unsatisfactory with the development of resistance. Relapse due to drug resistance or chemoresistance is a serious clinical problem frequently encountered in the TNBC patients. One of the main reasons for the relapse is attributed to the presence of a small subset of cells in the tumor called breast cancer stem‐like cells (BCSCs) or tumor‐initiating cells (TICs). BCSCs play a paramount role in tumor initiation, progression, and metastasis.^1^, ^2^, ^3^ In terms of resistance to therapy, BCSCs impart either constitutive or acquired resistance to chemotherapeutics or radiotherapy, which leads to poor prognosis.^4^ After the administration of the standard‐of‐care NACT against TNBC, which relies on actively dividing cells, the BCSCs survive the therapy along with some stromal cells that constitutes the minimal residual disease (MRD).^4^, ^5^ MRD is usually not detected by routine, clinical imaging techniques as those rely on certain minimum number of cells to be detected. After cessation of the cytotoxic or radiotherapy, the BCSCs from MRD, under appropriate stimulatory conditions, proliferate and undergo multi‐lineage differentiation program, replenishing the whole heterogeneous tumor. Such relapsed tumors are highly aggressive and are usually less responsive to the previously employed chemotherapy. They also likely become cross‐resistant to structurally and functionally unrelated chemotherapeutics, resulting in multidrug‐resistant (MDR) tumor cells. The unresponsive or chemoresistant MDR tumors present a grave prognosis as they are highly metastatic in nature. The intrinsic or acquired drug resistance and tolerance by BCSCs may arise due to many virtues in BCSCs. One of them is their ability to express a family of enzymes, such as aldehyde dehydrogenases (ALDHs). ALDHs can detoxify the drugs through metabolic conversion to less harmful or harmless products. Energetic BCSCs, which are a subset of BCSCs, display a high ALDH expression and demonstrate an upregulated capacity to proliferate and grow in an anchorage‐independent manner.^6^, ^7^ The other virtue by which the BCSCs display chemoresistance is through their expression of the ATP‐binding cassette (ABC) drug transporters on their plasma membrane. ABC transporters are integral membrane proteins that bind to the chemotherapeutic drugs in the cytoplasm of the tumor cells and pump them out to the cell exterior. Indeed, the BCSCs constitutively express ABCG2 or breast cancer resistance protein (BCRP) and serve as one of the key markers that are employed to identify BCSCs.^8^ The other two frequently encountered drug transporters in BCSCs are ABCB1 or P‐glycoprotein and ABCC1. These ABC transporters and possibly other influx and efflux transporters that are co‐expressed, co‐localized, and have a substantial overlap in their functions may impart the ability to BCSCs to withstand inimical exposure to xenobiotics including chemotherapeutic drugs. This points a compelling need to develop more effective treatments for mTNBC patients and provides a strong rationale to target the BCSC compartment of the tumor or co‐target BCSCs and bulk tumor cells (non‐BCSCs) to overcome drug resistance in mTNBC.

A recent comprehensive review discusses various molecular targets in BCSCs that could be potentially targeted in combination with standard NACT. In particular, targeting various signaling receptors and their downstream mediators or effectors that would reduce stemness and overcome chemoresistance of BCSCs were described.^1^ Ideally, an effective strategy would be to target the BCSCs that reduce their cancer stemness or plasticity. Targeting such mechanisms underlying cancer stemness or plasticity may lead to a durable therapy response. Sridharan et al, have discovered a key vulnerable node in triple‐negative BCSCs; in that, they are dependent on eukaryotic translation initiation factors (eIFs), especially eIF4A1.^9^ They implicated a possible role for eIF4A1 in mediating drug resistance in their paclitaxel‐resistant TNBC model in vitro. eIF4A1 is an mRNA helicase that unwinds the classical secondary structures located at the 5′‐leader sequence of selected, vital oncogenic mRNAs^10^ (Figure 1). The eIF4A1‐facilitated translation of oncogenic mRNA repertoire leads to the synthesis of many oncoproteins, such as survivin or BIRC5, myeloid cell leukemia 1(MCL1), cyclin D1, cyclin D3, mucin‐1C (MUC‐1C), Rho kinase 1 (ROCK1), ADP ribosylation factor 6 (ARF6), and murine double minute 2/human double minute 2 (MDM2/HDM2) and ADP ribosylation factor 6 (AFR6), which are vital for tumor cell survival both at primary and metastatic sites, proliferation, migration, local invasion, metastasis, and chemoresistance.^11^, ^12^, ^13^, ^14^, ^15^, ^16^

**FIGURE 1:**
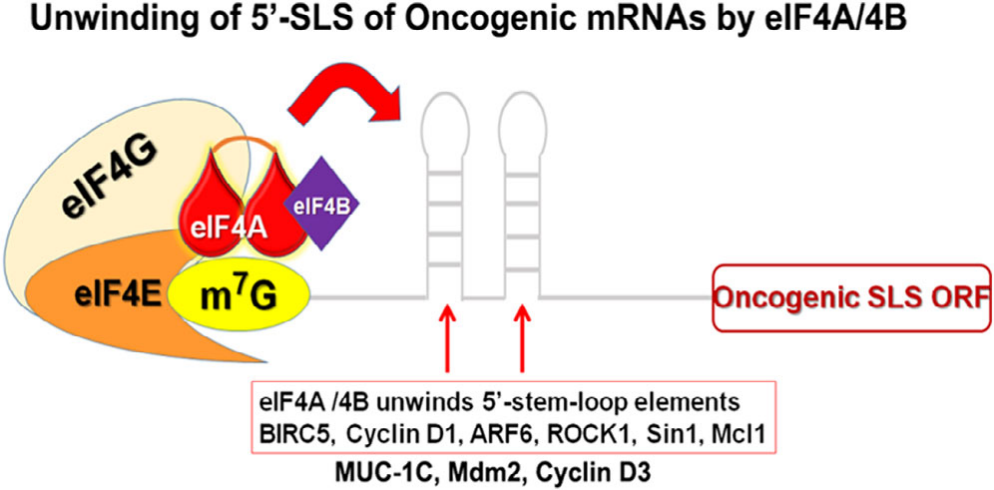
Unwinding of 5′‐leader sequence of oncogenic mRNAs by eIF4A1. eIF4A1 bound to the cap structure of the oncogenic mRNAs will unwind the classical secondary stem‐loop structures at the 5′‐leader sequence of oncogenic mRNAs. This will facilitate the facile scanning of the ribosome for the first AUG codon

Survivin plays a key role as a functional checkpoint for both mitosis and apoptosis in cancer cells; survivin and MCL1 are involved in chemoresistance as well.^17^ Cyclin D1 and cyclin D3 are also vital for clonogenicity and chemoresistance of the BCSCs..^10^, ^18^ Although nuclear cyclin D1 is known for its role in cell proliferation,^19^ the cytoplasmic cyclin D1 has a novel, non‐canonical role in cell migration.^20^, ^21^ Cyclin D1 activates CDK4/6, a current target in the clinics with palbociclib for chemoresistant forms of BC.^22^ ARF6 is one of the key proteins required for cell adhesion, migration, and invasion of cancer cells.^23^ ROCK1 promotes cell polarization and persistent directional migration (chemotaxis).^24^, ^25^ MDM2/HDM2, being an E3 ligase, can ubiquitinate wild‐type p53 and target it for degradation.^26^ In addition, perturbation of the chemokine GPCR, CXCR4, signaling promotes BC cell migration by regulating tumor cell adhesion events through provision of an optimal level of ROCK1 activity for effective cell migration.^27^ The chemokine receptor, CXCR4, has been demonstrated to activate Gα_i_/mTORC1 axis, which is upstream of eIF4A to promote spontaneous metastasis.^28^ Signaling from CXCR4 can also activate ribosomal S6 kinases—p90 ribosomal S6 kinase (p90^rsk^ ‐ via ERK pathway)^29^ and p70‐S6 kinase (p70^rsk^ ‐ via mTORC1 pathway) (Figure 2).^28^ These two major kinases feed into eFF4A by phosphorylating its endogenous inhibitor programmed cell death 4 (PDCD4) and targets it for degradation. This frees up some eIF4A from the PDCD4‐bound pool, which now can be incorporated into the eIF4F complex to initiate the cap‐dependent translation of oncogenic mRNAs.^30^ Interestingly, high level of expression of the chemokine receptor, CXCR4, in TNBC specimens predicts poor clinical outcome.^31^

**FIGURE 2:**
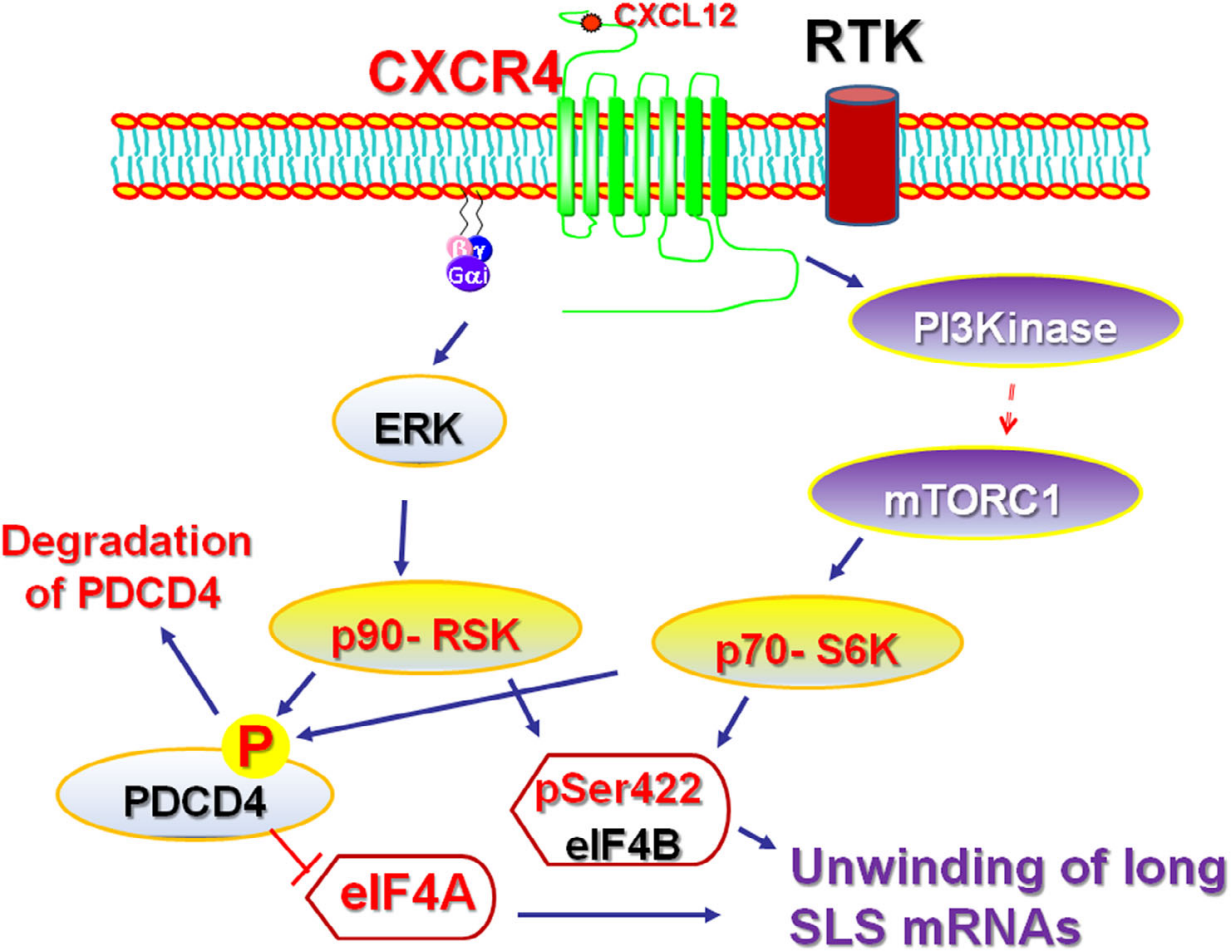
Signaling from G‐protein coupled receptors and receptor tyrosine kinases feed into activation of eIf4A1. The main signaling nodes, the phosphatidylinositol 3‐kinase (PI3K) and extracellular signal‐regulated (ERK) kinases, are activated by GPCRs, such as CXCR4, and receptor tyrosine kinases, such as epidermal growth factor receptor (EGFR) and human epidermal growth factor receptor 2 (HER2). PI3K and ERK subsequently activate ribosomal S6 kinases that phosphorylate the programmed cell death 4 (PDCD4) and mark it for degradation. This frees up the eIF4A1 to participate in the cap‐dependent protein translation. These pathways phosphorylate eIF4B, an ancillary protein that enhances the activity of eIF4A1, which speeds up the protein translation process

Through targeting this single mRNA helicase molecule, eIF4A1, it seems, the translation of a whole gamut of aforementioned oncogenic mRNAs can be inhibited. Sridharan et al, have demonstrated that some of the Yamanaka factors or transcription factors that regulate pluripotency or stemness or plasticity, such as OCT4, SOX2, and NANOG, were significantly downregulated when eIF4A1 was genetically ablated. These pluripotent transcription factors also cause drug resisatnce. A similar outcome was obtained when eIF4A1 was pharmacologically targeted with the natural, small molecule inhibitor, Rocaglamide A. This was the first report that highlights that targeting of eIF4A could downregulate all three pluripotency transcription factors that regulate BC stemness.^9^ Furthermore, the landmark finding is that when eIF4A1 is targeted by Rocaglamide A, the protein level of ABCB1 or P‐glycoprotein was significantly reduced. This was without any direct targeting of any of the ABC drug transporters. The mechanistic details as to how the targeting of eIF4A1 would reduce the BC stemness or diminish the protein levels of drug transporters remains to be elucidated. The interesting feature with targeting of eIF4A1 was equally effective in both therapy‐naïve and paclitaxel‐resistant TNBC cells. Furthermore, knocking out of the eIF4A1 in paclitaxel‐resistant TNBC cells reduced the pre‐existing resistance to paclitaxel dramatically. Overall, this brings a salient feature in that targeting eIF4A1 controls both BC stemness as well as drug resistance. Moreover, the stemness and chemoresistance are highly related to each other.^8^, ^9^ Importantly, the protein level of eIF4A1 is present in similar amounts between BCSCs and non‐BCSCs (bulk tumor cells). So, when eIF4A1 is targeted, both cellular populations will perish at the same time with less chance for MRD and tumor relapse. Thus, eIF4A1 is an actionable novel molecular target in the BCSC compartment, and controlling the helicase activity of eIF4A1 may lead to a favorable outcome in clinical chemoresistant cases of TNBC. Currently, Effector Therapeutics (NCT04092673) is recruiting patients for a phase I‐II clinical trial for targeting eIF4A1 with their synthetic small‐molecule inhibitor eFT226, which is somewhat analogous to Rocaglamide A.

As BCSCs play a role in drug tolerance and resistance, targeting the plasticity may lead to a profound and more durable clinical response. Targeting eIF4A1 seems a promising strategy to overcome TNBC stemness and chemoresistance in *in vitro* systems. A combinatorial treatment approach comprehensively targeting stem‐like cells may overcome the MDR encountered in the clinic and may result in a better objective treatment response in mTNBC.

## ETHICAL STATEMENT

Not applicable

## CONFLICT OF INTEREST

The authors have stated explicitly that there are no conflicts of interest in connection with this article.

## AUTHOR CONTRIBUITIONS

All authors had full access to the data in the study and take responsibility for the integrity of the data and the accuracy of the data analysis. *Conceptualization*, D.R., A.K.T.; *Data curation*, D.R.; *Funding acquisition*, D.R., A.K..; *Supervision*, D.R.; *Visualization*, D.R.; *Writing original draft*, D.R., A.K.T.; *Writing review editing*, D.R., A.K.T.; *Project administration*, D.R., A.K.T.

## Data Availability

Data sharing is not applicable to this article as no new data were created or analyzed in this study.
